# DNA Interaction Studies of Selected Polyamine Conjugates

**DOI:** 10.3390/ijms17091560

**Published:** 2016-09-19

**Authors:** Marta Szumilak, Anna Merecz, Malgorzata Strek, Andrzej Stanczak, Tadeusz W. Inglot, Boleslaw T. Karwowski

**Affiliations:** 1 Department of Hospital Pharmacy, Faculty of Pharmacy, Medical University of Lodz, 1 Muszynskiego Street, 90-151 Lodz, Poland; andrzej.stanczak@umed.lodz.pl; 2 Food Science Department, Faculty of Pharmacy, Medical University of Lodz, 1 Muszynskiego Street, 90-151 Lodz, Poland; anna.merecz@umed.lodz.pl (A.M.); boleslaw.karwowski@umed.lodz.pl (B.T.K.); 3 Department of Nucleic Acids Biochemistry, Medical University of Lodz, 251 Pomorska Street, 92-213 Lodz, Poland; malgorzata.strek@umed.lodz.pl; 4 Department of Medicinal Chemistry, Medical University of Lublin, 4 Jaczewskiego Street, 20-090 Lublin, Poland; tadeusz.inglot@umlub.pl

**Keywords:** intercalation, polyamine conjugates, chromone, quinoline

## Abstract

The interaction of polyamine conjugates with DNA double helix has been studied. Binding properties were examined by ethidium bromide (EtBr) displacement and DNA unwinding/topoisomerase I/II (Topo I/II) activity assays, as well as dsDNA thermal stability studies and circular dichroism spectroscopy. Genotoxicity of the compounds was estimated by a comet assay. It has been shown that only compound **2a** can interact with dsDNA via an intercalative binding mode as it displaced EtBr from the dsDNA-dye complex, with *K*_app_ = 4.26 × 10^6^ M^−1^; caused an increase in melting temperature; changed the circular dichroism spectrum of dsDNA; converted relaxed plasmid DNA into a supercoiled molecule in the presence of Topo I and reduced the amount of short oligonucleotide fragments in the comet tail. Furthermore, preliminary theoretical study has shown that interaction of the discussed compounds with dsDNA depends on molecule linker length and charge distribution over terminal aromatic chromophores.

## 1. Introduction

Due to the biological significance of DNA double helix in cell growth and proliferation, drugs that target DNA and its associated processes e.g., replication, transcription and translation inevitably dominate in chemotherapy regimens [1]. Lots of research projects are aimed at designing new entities capable of selectively interacting with the double stranded DNA (dsDNA) of tumor cells [2]. Among DNA recognizing small molecules, intercalators constitute a very important group of potential anticancer drugs [3]. They interact reversibly with DNA double helix by insertion of planar, aromatic chromophore between adjacent base pairs at the intercalation site which leads to topological changes in the double helix (unwinding, lengthening). Distorting DNA spatial conformation disrupts many DNA-protein interactions and, consequently, can cause cell death [4]. Representative monointercalators with antitumor activity e.g., doxorubicin, are still valuable drugs. Unfortunately, their therapeutic efficacy is often diminished by lack of selectivity, resulting in severe adverse reactions as well as the development of drug resistance [5]. In order to overcome these problems, bisintercalators have been designed [6]. They have unique structural features characterized by the presence of two planar polyaromatic or heteroaromatic systems joined by linker chains of different length and rigidity [7,8,9]. Such compounds can bind to dsDNA by bisintercalation which causes much more pronounced alterations in double helix structure due to simultaneous intercalation at two sites. Moreover, it results in higher dsDNA affinity and sequence specificity in comparison to monointercalating agents [10]. The complete structural requirements for bisintercalators to exhibit antitumor activity still remain not fully explained and vary depending on the characteristics of both terminal ring systems and the type of the linker chain [11]. The latter is very often a polyamine derivative [12]. Naturally occurring polyamines are vital in regulating cellular processes including proliferation, differentiation and apoptosis. The molecular mechanism of their action involves direct binding to DNA and modulation of DNA-protein interactions [13]. As organic multivalent cations, polyamines exert DNA-binding ability through reversible electrostatic interactions between the positive charges of polyamine and the negative charges of DNA [14]. It was elucidated that biogenic polyamines bind to major and minor grooves as well as phosphate groups, while guanine and backbone phosphate groups are major targets for synthetic polyamine analogues [15]. It is also known that polyamine binding to DNA depends on the distance between the positive charges on the polyamine relative to distance between the negative charges on DNA phosphate groups [16]. The regiochemical distribution of positive charge along the polyamines plays a major role in the condensation of DNA [17]. In addition, polyamine analogues with higher valency are more efficacious than spermine in provoking DNA condensation [18]. Moreover, direct measurement of free energy change involved in DNA condensation for different polyamines showed that the equilibrium free energy of the attractive component was always twice that of the repulsive component which suggested that DNA aggregation and condensation could spontaneously occur in the presence of multivalent polyamines [19,20].

The ability of polyamines and their analogues to promote conformational changes within DNA and its condensation to nanoparticles can facilitate the transport of oligonucleotides through the cell membrane and be helpful in developing simple carrier systems for gene delivery [17,21,22,23].

Rapidly dividing tumor cells require large amounts of polyamines. As a consequence, increased uptake of extracellular polyamines into tumor cells can be observed [24] and gives the opportunity to recognize a polyamine transport system as a chance for selective drug delivery [25]. Due to the above, polyamine analogues were designed. They were sufficiently similar in structure to the parent compound to be recognized by cancer cells as polyamine-like but unable to substitute their functionality, which more effectively induced growth arrest and apoptosis [26,27]. It has also been proposed that cytotoxic drugs acting through direct interaction with DNA can be covalently linked to polyamine vectors and targeted more selectively to cancer cells, see [16] for a review. 

Our quest for new anticancer drugs is focused on symmetrical compounds with bicyclic terminal chromophores designed in agreement with the bisintercalators’ structural requirements. We decided to link chromophore rings by an aminoalkyl chain as this type of linker is positively charged at a physiological condition and can interact with negatively charged phosphate groups of DNA [19,23]. Therefore, such a DNA binding linker could enhance affinity of bis-chromophore molecules to DNA and promote the formation of an intercalation complex by placing flat pendant moieties in an optimal position for stacking interactions [28]. This concept was used earlier by several other authors who obtained bisintercalators with higher DNA binding constants when compared to corresponding monointercalators [3,29]. 

**Figure 1:**
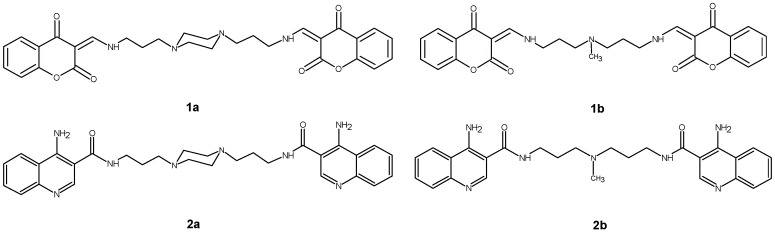
Chemical structure of examined compounds.

It has been shown that dimeric molecules with chromone (**1b**) and quinoline (**2a**) scaffolds (Figure 1) are promising entities due to their drug-like properties [30] and antiproliferative activity toward highly aggressive melanoma cell line A375, prostate cancer cell lines PC-3, DU-145 and breast cancer cell line MCF-7. The IC_50_ values for the most active derivative **2a** were in the range of 16.8 to 26.6 µM. In addition, **2a** induced programed cell death in prostate and breast cancer cell lines by intrinsic pathway, which involved depolarization of mitochondria together with disruption of its membrane [31,32,33]. Our studies revealed that anticancer effectiveness of this group of compounds strongly depends on cancer cell line type [31,32,33] but these differences may also have their origin in the way the compounds bind to double helix. Therefore, expanding our knowledge about the possible interaction of these compounds with dsDNA or its associated targets such as topoisomerases is of equal importance. In addition, it is an indispensable part of the rational drug discovery process which will allow to obtain new effective substances for cancer therapy.

Based on previous data, it can be postulated that the biological activity of polyamine derivatives with bicyclic chromophores depends on the differences in their spatial structure [31,32,33]. Due to the fact that these compounds have been designed as potential intercalators, further studies are focused on the explanation of the influence of both molecule subunits, namely terminal aromatic rings, and the linker on the dsDNA binding properties. Therefore, four symmetrical compounds with different combinations of heteroaliphatic linker and terminal bicyclic systems have been chosen **1a**, **1b**, **2a**, **2b** (Figure 1). The following experimental methods [34], useful in distinguishing the binding mode of molecular ligands, i.e.,: ethidium bromide (EtBr) displacement and DNA unwinding/topoisomerase I/II (Topo I/II) activity assays as well as the analysis of dsDNA thermal stability (*T*_m_-melting temperature) and circular dichroism (CD) spectroscopy have been performed. Moreover, the genotoxicity of examined polyamine conjugates was evaluated by the comet assay.

## 2. Results

### 2.1. Ethidium Bromide Displacement Assay

**Figure 2:**
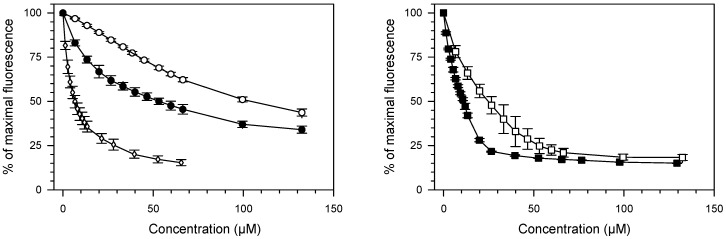
Ethidium bromide (EtBr) displacement assay. Changes of EtBr-calf thymus DNA complex fluorescence observed after the addition of increasing amounts of tested compounds are displayed. (●) **1a**; (○) **1b**; (◊) 9-aminoacridine (**9AA**); (■) **2a**; (□) **2b**. Points represent means of three experiments ± Standard deviation (S.D).

Due to the fact that tested compounds contain planar rings in their structures, it is possible that they may interact with DNA by the formation of an intercalation complex [35]. To check this possibility, a DNA intercalation assay was performed, using 9-aminoacridine (**9AA**) as a reference intercalator (see Figure S2). As shown in Figure 2, compounds **1a**, **1b**, **2a**, **2b** caused the reduction of EtBr/DNA complex fluorescence. This result indicates that all tested compounds compete with EtBr for binding sites and displace this dye from the DNA-drug complex.

Compounds containing quinoline rings **2a** and **2b** were found to be more potent DNA binders than their analogues **1a** and **1b** with chromone moieties. C_50_ values for **2a** and **2b** were 2.81 ± 0.53 µM and 11.10 ± 0.36 µM, respectively. C_50_ values for **1a** and **1b** were found as follows: 52.9 ± 3.18 µM and 104.67 ± 5.01 µM. Apparent DNA binding constants for **2a** and **2b** were higher by approximately one order of magnitude when compared with that of **1a** and **1b** (Table 1). Moreover, DNA binding parameters of tested compounds depend, not only, on the ring structure but also on the structure of a linker. In each pair of analogues, higher affinity to dsDNA (lower C_50_ and higher *K*_app_) was found for compounds possessing less flexible linker (Table 1). The most potent dsDNA binder, among all tested compounds, was **2a** which was the only one compound exhibiting a higher affinity to double helix, than the reference compound **9AA**.

### 2.2. Thermal Melting Studies

Compounds with DNA binding properties may stabilize the DNA duplex and increase oligonucleotide melting temperature (*T*_m_), due to the complementary strands separation prohibition [37]. Examined compounds **1a**, **1b**, **2a**, **2b** were tested at a concentration of 15 μM after oligonucleotide strands hybridization (Table 2). Due to the fact that compound **2a** showed higher thermal stability than the reference ds–nucleotide, it can be concluded that **2a** induces some stabilization effects on the DNA double helix. Other examined compounds do not affect the thermal stability of DNA (Table 2).

### 2.3. DNA Unwinding Assay

Topo I relaxes supercoiled plasmid by causing single-strand breaks and religation. However, the presence of an intercalative agent induces additional negative supercoils and compensatory positive superhelical twists in circular DNA. It has been reported, that relaxed plasmid treated with Topo I and intercalative agent is converted to negatively supercoiled [38,39].

**Figure 3:**
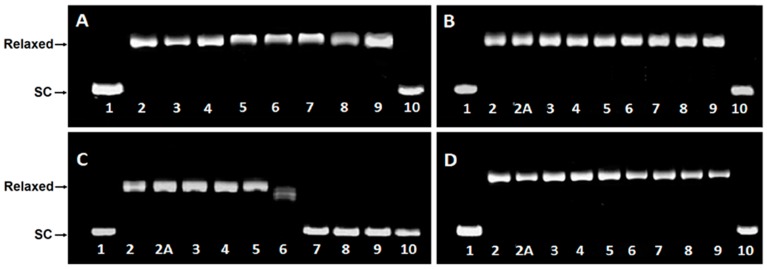
Influence of compounds **1a** (**A**), **1b** (**B**), **2a** (**C**) and **2b** (**D**) on conversion of relaxed plasmid DNA to supercoiled molecule. Control reactions were carried out in the absence of Topo I (supercoiled plasmid, SC) (lane 1), with Topo I (relaxed plasmid) (lane 2), with Topo I and 0.1% DMSO (lane 2A). Plasmid conformation was analyzed in increasing concentrations of investigated compounds (lane 3–9, concentration: 0.5; 1; 5; 10; 15; 20 and 30 µM, respectively) with constant Topo I concentration. **9AA** (100 µM) was used as positive control (lane 10).

DsDNA binding properties of compounds **1a**, **1b**, **2a**, **2b** are presented in Figure 3A–D. Supercoiled DNA is fully relaxed in the presence of Topo I and in the absence of the compound. It has been observed that **2a**, at the concentration >10 µM in the presence of Topo I, converted relaxed plasmid to supercoiled molecule, whereas the relaxation process has been still observed in the presence of compounds **1a**, **1b** and **2b** even in a 30 µM drug concentration.

### 2.4. Topoisomerase I Activity Assay

**Figure 4:**
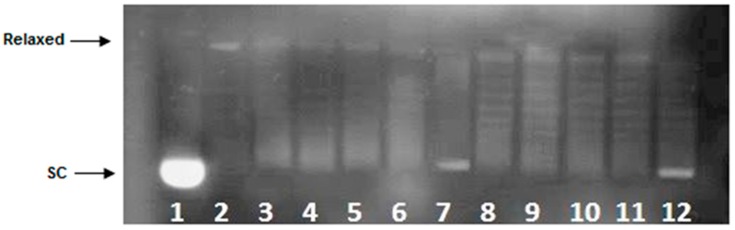
Influence of **2a** on Topo I activity. The rate at which relaxed plasmid DNA was converted to supercoiled molecule was monitored over a time course of 30 min. Lane 1—supercoiled plasmid, SC; lane 2—plasmid with Topo I after 30 min of incubation time; lane 3–7—plasmid with Topo I and in the presence of compound **2a** at the concentration of 15 μM after following incubation times: 1; 5; 10; 15; 30 min, respectively; lane 8–12—plasmid with Topo I and **9AA** at the concentration of 100 µM after following incubation times: 1; 5; 10; 15; 30 min, respectively (positive control).

To further elucidate the mechanism of **2a** interaction with dsDNA, its influence on the Topo I activity was evaluated when tested at a constant concentration of 15 µM. As can be seen in Figure 4, supercoiled DNA is fully relaxed in the presence of Topo I and in the absence of the examined compound. It has been observed that **2a** at the concentration of 15 µM did not inhibit the overall activity of Topo I as relaxed plasmid was converted to negatively supercoiled after 30 min of incubation time.

### 2.5. Topoisomerase II DNA Cleavage Assay

**Figure 5:**
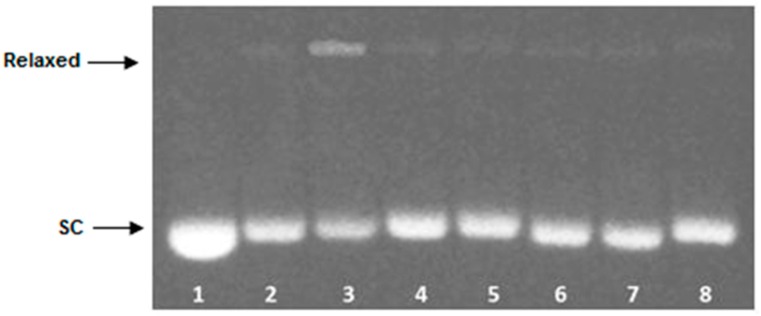
Influence of **2a** on Topo II activity. The Topo II-mediated plasmid cleavage assay was carried out over constant enzyme concentration. Lane 1: supercoiled plasmid, SC; lane 2: plasmid with Topo II after 15 min of incubation time; lane 3: plasmid with Topo II and etoposide (50 µM) after 15 min of incubation time (positive control); lane 4–8: plasmid with Topo II and in the presence of compound **2a** at the concentration of 5, 10, 15, 20, 30 µM, respectively.

Topoisomerase II (Topo II) is an enzyme that catalyzes relaxation of supercoiled DNA by cutting both strands of a dsDNA molecule, passing one DNA helix through another, and resealing the cut in a process utilizing ATP [40]. Topo II is the target for several potent anticancer drugs e.g., etoposide (see Figure S2) that generate high levels of enzyme-DNA cleavage complexes [41]. Enhanced cleavage complex formation results from the ability of etoposide to inhibit DNA religation and stimulate enzyme-linked DNA breaks which induce cell death pathway [42]. To determine whether **2a** enhances the Topo II-mediated DNA cleavage, the effect on DNA scission mediated by aforementioned enzyme was examined. As can be seen in Figure 5, compound **2a** did not target Topo II catalytic activity as enzyme-mediated DNA strand breaks induced by **2a** was not observed. 

### 2.6. Comet Assay

**Figure 6:**
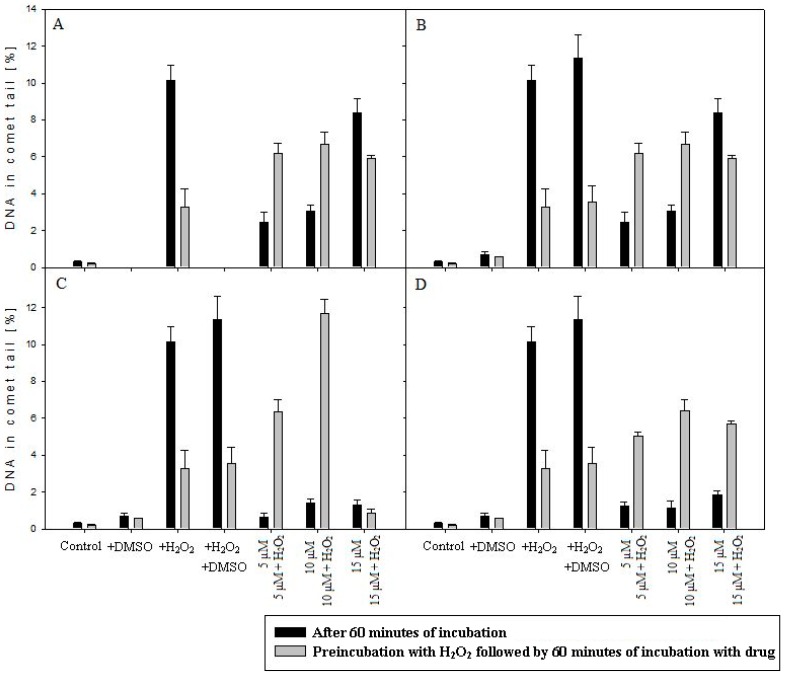
DNA damage in leukemia cells induced by **1a** (**A**), **1b** (**B**), **2a** (**C**) and **2b** (**D**) (60 min, 37 °C) at concentrations of 5, 10 and 15 µM with and without prior H_2_O_2_ incubation (10 min, 4 °C) at a concentration of 15 µM with respect to the appropriate control (−H_2_O_2_/+H_2_O_2_). The values were measured as the average percentage of DNA in the comet tail ± SEM using alkaline version of comet assay.

Comet assay was conducted in order to determine the level of DNA damage after incubation with tested compounds. Figure 6A–D shows DNA damage in leukemia cells (MOLT-4) treated with **1a**, **1b**, **2a**, **2b**.

The results of the comet assay indicated that the level of DNA damage, after H_2_O_2_ treatment at a concentration of 15 μM was higher (*p* < 0.001) than in control without damaging agent. Moreover, compounds **1a**, **1b**, generated DNA damage in the absence of H_2_O_2_ in a concentration-dependent manner. The increase of DNA damage generated by **2a** and **2b** at a concentration of 5, 10, 15 µM, in the absence of pre-incubation with H_2_O_2_, was not observed (the increase was not statistically significant) (Figure 6C,D). However, in the presence of **2a** (at a concentration of 15 µM) after prior incubation with H_2_O_2_, the decrease of DNA damage was denoted (Figure 6C, Figure S3). Therefore, it can be postulated that **2a** with 1,4-*bis*(3-aminopropyl)piperazine as the linker may reduce considerably the effectiveness of the DNA migration, after lesion induction, due to insertion of molecules between stacked DNA base pairs. Contrary to that, compound **2b** without piperazine moiety in the linker, under the same experimental condition, caused an increase in the percentage of short DNA fragments in the comet tail (Figure 6D). The same pattern of DNA lesion in the comet tail was denoted for compounds **1a** and **1b** (Figure 6A,B).

### 2.7. Circular Dichroism Spectroscopy

**Figure 7:**
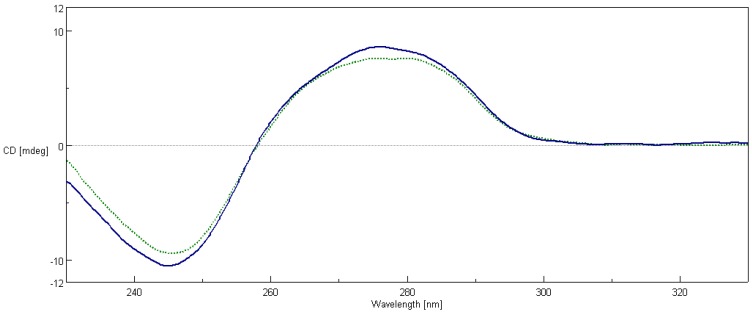
The circular dichroism spectra of pure ct-DNA 100 µM (solid) and ct-DNA incubated with **2a** at concentration of 10 µM (dots).

The CD spectroscopy is a useful method to assess changes in DNA conformation during ligand-DNA interactions. Calf thymus DNA (ct-DNA) exhibits two conservative CD bands, a positive band at 277 nm due to π–π base stacking and a negative band at 245 nm due to right handed helicity which is characteristic of DNA in the right-handed B form [43,44]. These bands are sensitive towards the binding of molecules; hence CD spectroscopy is useful in monitoring the conformational variations of DNA in the presence of the studied compound [45]. The CD spectra of the ct-DNA and ct-DNA/**2a** mixture in the Ultraviolet (UV) region were recorded. As shown in Figure 7, the positive (276 nm) and negative (245 nm) bands were reduced significantly in intensity, suggesting that compound **2a** induced disturbance on DNA base stacking and DNA right-handed helicity, mainly due to its aromatic planarity. The decreases in the intensities of both positive and negative bands can usually be observed in the intercalative binding of small molecules to DNA [46]. The characteristics of the B-DNA form CD spectrum is still conserved and the arrangement of the DNA bases is altered.

**Figure 8:**
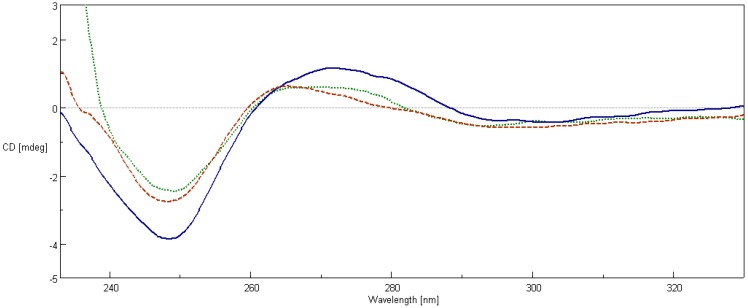
The CD spectra of pure ds-oligonucleotide 2.5 µM (solid), ds-oligonucleotide with **2a** at a concentration of 2.5 µM added before hybridization (ON1—dots) and ds-oligonucleotide with **2a** at a concentration of 2.5 µM added after hybridization (ON2—dashes).

Figure 8 shows the CD spectra of ds-oligonucleotide before and after the addition of compound **2a**. Changes include the reduction in the negative value at 248 nm, the decrement in the peak at 270 nm, and a slight blue shift in ON2 band at 270 nm. Similarly to changes in CD spectra of ct-DNA, we can assume that the addition of **2a** caused a disturbance to π–π base stacking and the right-handed helicity of double stranded oligonucleotide. Comparison between the CD spectra of ON1 and ON2 mixtures with added **2a** shows that the intercalation effect occurred regardless of whether a ligand was added before or after hybridization of oligonucleotides.

## 3. Discussion

Our studies on polyamine conjugates with bicyclic terminal chromophores revealed that the most promising molecules are quinoline and chromone derivatives, exhibiting drug-like properties [30]. The representative compounds were active against melanoma cell line A375, prostate cancer cell lines PC-3, DU-145 and breast cancer cell line MCF-7. IC_50_ values for the most active derivative **2a** were in the range of 16.8 to 26.6 µM depending on cancer cell line. In addition, **2a** induced programed cell death in prostate and breast cancer cell lines by an intrinsic pathway, which involved depolarization of mitochondria together with disruption of its membrane [31,32,33]. The design of effective anticancer drugs requires not only the biological activity assessment but also an understanding of the mechanisms involved in the process of eliminating cancer cells. Discussed compounds were intended to act as bisintercalators, therefore we decided to perform experiments evaluating their ability to interact with double stranded DNA. From a variety of techniques, which are used to establish dsDNA binding properties of small molecules [34], EtBr displacement assay, thermal stability studies, DNA unwinding/topo I/II activity assays and circular dichroism have been chosen. Preliminary studies using the EtBr displacement assay indicated that polyamine conjugates bind to DNA with apparent binding constants in the range of 4.26 × 10^6^ M^−1^ to 0.11 × 10^6^ M^−1^ (Table 1). The fact that examined compounds have the ability to displace EtBr from DNA-dye complex (Figure 2) suggests that they may intercalate into double helix [35]. Possibly, the most favourable structure for intercalation into DNA is **2a**, as this compound exhibited the highest DNA binding parameters exceeding well-known intercalator 9-aminoacridine (**9AA**). It is not clear whether other tested compounds are able to form intercalative complex with dsDNA, as their binding parameters were significantly lower than that assigned for **2a** (Table 1). Moreover, it is possible that the reduction of fluorescence of the EtBr-DNA complex was caused by aminoalkyl linkers binding to DNA instead of the intercalation process. Such an effect of polyamines was reported by Vijayanathan et al. [17] who also used a EtBr displacement assay to measure the affinity of several polyamines to DNA. It is known that polyamines which are not intercalating compounds, induce changes in DNA double helix structure leading to DNA condensation and aggregation [19,23]. Structural changes accompanying these processes seem to destabilize the intercalation complex of EtBr with DNA. As a consequence, EtBr displacement assay is not sufficient to prove the intercalative binding of tested compounds to DNA. Therefore, dsDNA melting studies and topoisomerase assays were used to more definitively establish the nature of interactions between discussed polyamine conjugates and dsDNA. The analysis of *T*_m_ values has shown that only compound **2a** increased dsDNA stability by 7 °C in comparison to the control. For other discussed compounds (**1a**, **1b**, **2b**) the changes of melting temperatures were negligible (Table 2). It is important to mention, that for reference compound **9AA**, *T*_m_ = 78 °C was denoted. Based on the above data and principal rules governed to the intercalation process, it can be postulated that only compound **2a** can be recognized as a potential intercalator. These findings were confirmed by a DNA unwinding assay in which only compound **2a**, at the concentration >10 µM in the presence of Topo I, converted relaxed plasmid to negatively supercoiled DNA molecule (Figure 3C), whereas the relaxation process has still been observed in the presence of compounds **1a**, **1b** and **2b** even in 30 µM drug concentration (Figure 3A,B,D). This result has been supported by a Topo I activity assay (Figure 4), which revealed that **2a** did not inhibit the catalytic activity of Topo I. Contrary to that, in the absence of **9AA** or **2a** the relaxation of supercoiled DNA by Topo I is not disturbed. As far as the influence on Topo II activity is concerned, **2a** did not induce an increase in Topo II-mediated DNA cleavage (Figure 5). Circular dichroism analysis has confirmed the intercalation of compound **2a** to ct-DNA due to a reduction of the CD spectrum intensity simultaneously in the two areas at 245 and 277 nm. CD analysis of **2a** intercalation to ds-oligonucleotide gave similar results.

Due to this fact, that the described results have been obtained for synthetic or isolated ds-oligonucleotide, we have decided to perform the comet assay experiment on leukemia cell line (MOLT-4) to check the binding ability of discussed compounds to genome. As shown in Figure 6, only compound **2a** considerably reduced the effectiveness of the DNA migration, after lesion induction, probably due to the insertion of molecules between stacked DNA base pairs. For other discussed compounds, the percentage of DNA damage induced by H_2_O_2_ was at the same concentration level. The difference between **2a** and **1a**, **1b**, **2b** can be explained as the result of bisintercalative binding mode of **2a** which “stapled” damaged DNA fragments (“zipper like effect”). To elucidate this phenomenon, the simply 3D structures optimization has been performed (Density functional theory (DFT), B3LYP/6-31G in gaseous phase). Obtained results showed that, in the case of molecules **1b** and **2b** with 3,3′-diamino-*N*-methyldipropylamine as the linker, the distance between terminal nitrogen atoms N1 and N2 (see Supplementary Materials Figure S1) was shorter than in **1a** and **2a** derivatives with piperazine as the central subunit (see Table S1, Figure S1). Moreover, the presence of a N-CH_3_ group in the center of molecules **1b**, **2b** forced their structure linearization. In contrast, replacing this group by a piperazine ring (**1a**, **2a**) bended the molecules’ spatial geometry. The interaction of **2a** with dsDNA may be additionally enhanced by the electrostatic interaction between positively charged piperazine nitrogen atoms and DNA backbone phosphate group. Finally, to determine the charge distribution, natural population analyses (NPA) with natural bond order (NBO) were performed using the B3LYP functional and 6-31G basis set. As shown in Table S1, the difference in charge distribution over aromatic terminal rings was denoted for compounds **2a**, **2b**, (−0.190/0.600 and −0.169/0.618, respectively), while for molecules, **1a** and **1b** almost the same negative value is retained (−0.647/−0.634 and −0.642/−0.637, respectively).

## 4. Materials and Methods

### 4.1. Examined Compounds

Compounds **1a**, **1b**, **2a** and **2b** used in this study were selected from previously synthesized and in vitro evaluated polyamine derivatives on the basis of their diverse biological activity and proper chemical structure. Their synthesis and analytical data were described earlier [31,32]. Compound **1a** was dissolved in water whereas compounds **1b**, **2a**, **2b** were dissolved in dimethyl sulfoxide DMSO (final concentration was 0.1% in all samples).

### 4.2. Ethidium Bromide Displacement Assay

To check whether tested compounds may interact with DNA, an EtBr displacement assay was carried out according to the method described by Cain et al. [35]. A 3 mL buffer containing 2 mM Hepes, 0.01 mM EDTA, 9.4 mM NaCl 1.26 µM EtBr, pH 7.0 was mixed in a fluorescence cuvette with 1 µL of 1 mg/mL solution of calf thymus DNA (Worthington, Lakewood, NJ, USA). The cuvette was placed in a fluorescence spectrophotometer (Perkin-Elmer LS55, Waltham, MA, USA). Then 1 µL portions of compound solutions (20 mM in DMSO) were added step by step and after thorough mixing the intensity fluorescence was read (Ex 546 nm, Em 595 nm). This method is based on the competition of an added compound with EtBr for DNA intercalation sites. The fluorescence intensity of EtBr increases upon DNA binding. The addition of a compound displacing intercalated EtBr leads to quenching the fluorescence caused by the EtBr/DNA complex. The percent of fluorescence decrease was plotted against the concentration (µM) of each compound and C_50_ value of each was determined. C_50_ is defined as the concentration of the added compound required to reduce the fluorescence of the EtBr/DNA complex to 50%. C_50_ values were used to calculate apparent binding constants (*K*_app_) of tested compounds to DNA [36].

### 4.3. Thermal Melting Studies

In this study the following oligonucleotides:

1: 5′-AAATTAATATGTATTGTATATAAATTATT-3′
2: 3′-TTTAATTATACATAACATATATTTAATAA-5′

were employed. Oligonucleotides were purchased as HPLC-purified compounds from the Bioorganic Chemistry Department, Polish Academy of Science, Lodz, Poland (Geneworld synthesizer, K&A Laborgeraete GbR, Schaafheim, Germany) using nucleotide phosphoroamidites synthons as substrates (ChemGenes Corporation, Wilmington, MA, USA).

The hybridization was carried out in a reaction volume of 1 mL containing single stranded oligonucleotide 1 and 2, 0.1 M NaCl, 0.01 M MgCl_2_, by heating to 90 °C for 10 min followed by slow cooling to room temperature in the presence or absence of different drug concentrations. In this study, the following compounds were employed: **1a**–**b**, **2a**–**b** (15 µM), DMSO control (final concentration was 0.1%) and 9-Aminoacridine hydrochloride hydrate (**9AA**) (Sigma-Aldrich, Saint Louis, MO, USA) (100 µM) as a positive control. The DNA melting point was determined spectrophotometrically on Cary 1.3E UV-Vis spectrophotometer (Agilent Technologies, Santa Clara, CA, USA) by using a computer equipped with Cary WinUV software (Agilent Technologies: Santa Clara, CA, USA). The absorbance changes at 260 nm was measured every minute in the range of 21 °C to 80 °C with an increment of 1 °C/min and 1 min as equilibration time. *T*_m_ values were obtained from the midpoint of the first-derivative plots. Studies were repeated two times [37].

### 4.4. DNA Unwinding Assay

#### 4.4.1. Strains and Media

*Escherichia coli* DH5α cells with the plasmid pENTR4 were supplied from the Pharmaceutical Biotechnology Department, Medical University of Lodz. A Luria Broth (LB) medium (10 g tryptone, 5 g yeast extract, 2 g glucose and 10 g NaCl per liter of medium) was used for the growth of all cultures.

#### 4.4.2. Bacterial Culture and Plasmid Isolation

Agar plate supplemented with kanamycin (30 μg/mL) was inoculated with *E. coli* DH5α containing pENTR4 plasmid and incubated overnight, at 37 °C. The bacterial colonies were resuspended and subsequently, 250 mL of an LB medium supplemented with kanamycin (30 μg/mL) was inoculated with the overnight culture equivalent to the 0.5 McFarland. The culture was incubated for 13 h at 37 °C with vigorous shaking (150 rpm). Plasmid was isolated from bacteria using a Plasmid Mini DNA purification system (A & A Biotechnology, Gdynia, Poland) as described by the manufacturer. Then, the supercoiled form was isolated from agarose gel using a Gel-Out Kit (A & A Biotechnology, Gdynia, Poland) as described by the manufacturer.

DNA unwinding assay was carried out according to the method described by Sappal et al. [39] with a few modifications. Supercoiled pENTR4 DNA (0.2 μg) was a substrate for the reaction. Bacterial Topo I was expressed in an Escherichia coli strain containing the cloned topA gene (New England Biolabs, Ipswich, MA, USA). Plasmid was incubated with 2 units of Topo I in reaction to the volume of 20 μL (10 mM Tris-HCl (pH 7.5), 175 mM KCl, 5 mM MgCl_2_, 0.1 mM EDTA and 2.5% glycerol) in the presence of varying concentrations of the drug under study: **1a** (0.5–30 µM), **1b** (0.5–30 µM), **2a** (0.5–30 µM), **2b** (0.5–30 µM), DMSO control (final concentration was 0.1% in all samples) and **9AA** (100 µM) as a positive control. Reactions were started after the addition of the enzyme and stopped after 60 min at 37 °C by extracting the plasmid DNA with phenol-chloroform (*v*/*v*) followed by adding a stop solution (0.77% SDS, 0.77 mM EDTA (pH 8.0). Samples were then added to an electrophoresis dye mixture (Polgen, Lodz, Poland), loaded onto 1% agarose gel running 1.5–2 V/cm in a TAE buffer (40 mM Tris-acetate, pH 8.5 and 10 mM EDTA). The gels were stained with EtBr 0.5 μg/mL, observed under UV light (at 260 nm) and photographed using a Gel Doc system (Syngene, Cambridge, UK).

### 4.5. Topoisomerase I Activity Assay

Double stranded, closed circular pBR322 plasmid originated from Thermo Fisher Scientific (Waltham, MA, USA).

Topoisomerase I activity assay was carried out according to the method described by Sappal et al. [39] with a few modifications. Supercoiled pBR322 DNA plasmid was incubated with six units of Topo I in a reaction volume of 20 µL (50 mM Potassium Acetate, 20 mM Tris-acetate, 10 mM Magnesium Acetate, 100 μg/mL BSA, pH 7.9) in the presence of **2a** (15 µM) and **9AA** (100 µM) as a positive control. The reactions were started after the addition of the enzyme and stopped up to 30 min (five time points: 1, 5, 10, 15 and 30 min) at 37 °C by extracting the plasmid DNA with phenol–chloroform (*v*/*v*) following by adding stop solution (0.77% SDS, 0.77 mM EDTA, pH 8.0). Samples were then added to an electrophoresis dye mixture (Polgen, Lodz, Poland), loaded onto 1% agarose gel running 1.5–2 V/cm in a TAE buffer (40 mM Tris-acetate, pH 8.5, and 10 mM EDTA). The gels were stained with EtBr 0.5 µg/mL, observed at UV light (260 nm) and photographed using a Gel Doc system (Syngene).

### 4.6. Topoisomerase II DNA Cleavage Assay

A topoisomerase II DNA cleavage reaction was carried out according to the method described by Sappal et al. [39] with a few modifications. Supercoiled pBR322 DNA was incubated with four units of Topo II in a reaction volume of 20 µL (35 mM Tris-HCl, 24 mM KCl, 4 mM MgCl_2_, 2 mM DTT, 1.75 mM ATP, 5 mM spermidine, 0.1 mg/mL BSA, 6.5% glycerol, pH 7.5) in the presence of **2a** (5–30 µM) and etoposide (50 µM) as a positive control. The mixture was incubated for 15 min at 37 °C. After relative time has finished, the addition of 2 µL of 5% SDS and 1 µL of 375 mM EDTA, pH 8.0 followed by proteinase K treatment (digestion of proteinase K was performed in the presence of 2 µL of 0.8 mg/mL of protein and heated the mixture to 45 °C for 30 min) stopped the enzyme reaction. Samples were then added to an electrophoresis dye mixture (Polgen, Lodz, Poland), and loaded onto 1% agarose gel running 1.5–2 V/cm in a TAE buffer (40 mM Tris-acetate, pH 8.5, and 10 mM EDTA). The gels were stained with ethidium bromide 0.5 µg/mL, observed under UV light and photographed using a Gel Doc system (Syngene).

### 4.7. Comet Assay

MOLT-4 (acute lymphoblastic leukemia) cells were cultured in an RPMI-1640 medium supplemented with antibiotics (penicillin, streptomycin) and 10% fetal calf serum, in a 5% CO_2_-95% air atmosphere and at a temperature of 37 °C [47].

A comet assay was performed as described by Singh et al. [48] with a few modifications. To assess DNA damage, MOLT-4 cells were incubated at different concentration (5, 10, 15 µM) of the following compounds: **1a**, **1b**, **2a** and **2b** in the absence or presence of prior incubation with hydrogen peroxide (H_2_O_2_) to a final concentration of 15 µM (10 min on ice). A sample without compounds and a sample with DMSO (0.1%) were employed as a negative control, and a sample with H_2_O_2_ alone was employed as a positive control.

The cell suspensions (2 × 10^5^ cells) were embedded in 40 µL of 1% low melting point agarose and spread on a slide precoated with a 1% normal melting point agarose. Agarose cell suspensions were allowed to solidify at 4 °C and the slides were transferred to a lysis solution (2.5 M NaCl, 0.1 M EDTA, 10 mM Tris and 1% Triton X-100, pH 10) at 4 °C for 60 min, then slides were placed in an electrophoresis chamber exposed to an alkaline buffer (0.3 M NaOH and 1 mM EDTA, pH 13) for 20 min by unwinding of DNA. Then, electrophoresis was performed for 20 min at 21 V/30 mA and electrophoresis slides were neutralized and stained with 4′,6-diamidino-2-phenylindole (DAPI).

The stained nuclei were visualized by a fluorescent microscope under 40× magnification. Images of comets for analysis were obtained using a camera conjugated to a fluorescent microscope (Delta Optical, Minsk Mazowiecki, Poland). Slides were scored using an image analysis system, Casp (J. Kochanowski University, Kielce, Poland). Measurements were made for 50 cells per slide and the percent of DNA in comet tail was used as a quantitative measure of the DNA damage.

All the values in this study were expressed as mean ± S.E.M. Differences between mean values were tested using the *t*-test. *p*-value less than 0.05 was considered statistically significant. The data were analyzed using STATISTICA 6.0 software (Statsoft, Tulsa, OK, USA).

### 4.8. Circular Dichroism Spectroscopy

Circular dichroism measurements were carried out on a Jasco J-815 spectropolarimeter under the following conditions: temperature 37 °C, rectangular 1 cm quartz cell, range 230–330 nm, resolution 0.2 nm, scan speed 50 nm/min. 

The calf thymus DNA solution in a concentration of 100 µM was prepared in a TBS buffer containing 50 mM Tris-HCl, 150 mM NaCl, pH 7.4. The solution of ct-DNA gave a ratio of UV absorbance at 260 and 280 nm of 1.87, indicating that the DNA was free of protein contamination. The DNA concentration was determined by the UV absorbance at 260 nm using ε = 6600 M^−1^·cm^−1^. Ct-DNA was stabilized at 37 °C for 1 h and CD spectrum was measured. Next, 10 μL of **2a** solution (in DMSO-TBS 1:9 *v*/*v*) was added giving a molar concentration ratio of 1:10 with respect to ct-DNA. The solution was incubated in the measuring cell for 1 h at 37 °C and then the CD spectrum was obtained. The CD spectra of buffer and **2a** compound were checked for intrinsic CD effect, giving negative results.

The following oligonucleotides: 5′-AAATTAATATGTATTGTATATAAATTATT-3′ and 3′-TTTAATTATACATAACATATATTTAATAA-5′ were mixed in equimolar proportions in a TBS buffer, pH 7.4, containing 1 mM EDTA. Portions of 2.5 mL of the mixture were transferred to two quartz cuvettes (ON1 and ON2). An amount of 2.5 μL of the **2a** solution was added to an ON1 cuvette (to give a molar concentration ratio of 1:1 with respect to ds-oligonucleotide), then both were sealed and heated at 95 °C for 5 min. Subsequently, they were annealed by slowly cooling to 37 °C. Preliminary experiments showed that after incubation of oligonucleotides, the UV absorption at 260 nm decreased by 30%, which indicated a double-strand formation. Molar concentration of double-stranded oligonucleotide (2.5 µM) was determined using OligoCalc: Oligonucleotide Properties Calculator [49]. After stabilization in 37 °C, CD spectra of both solutions were measured. Afterwards, 2.5 μL of **2a** solution was added to a cuvette containing pure ds-oligonucleotide (ON2). The mixture was incubated in 37 °C for 1 h and CD spectrum was obtained.

### 4.9. Computational Methodology

The molecular geometries of neutral **1a**–**b**, **2a**–**b** in the gaseous phase were initially optimized by molecular mechanics using the universal force fields [50]. All subsequent calculations were performed by the density functional theory (DFT) using the generalized gradient approximation (GGA) exchange-correction functional, in which the B3LYP functional was implemented (Becke’s three-parameter hybrid HF/DFT exchange functional, and the Lee-Yang-Parr correlation functional) [51,52]. For all calculations, the 6-31G basis set was used [53]. For characterisation of the stationary point of all the investigated molecules, the harmonic vibration was calculated at the B3LYP/6-31G level, no imaginary frequencies have been found. To determine the charge distribution, natural population analyses (NPA) with natural bond order (NBO) were performed using the B3LYP functional and 6-31G basis set. The calculations of all the structures were achieved with Gaussian 09, Revision D.01 [54].

## 5. Conclusions

The experimental findings presented above clearly indicate that only compound **2a** has the ability to interact with a double helix via intercalative binding mode. It displaced EtBr from a DNA-dye complex and exhibited the highest DNA binding parameters, exceeding even well-known intercalator 9-aminoacridine (**9AA**). In addition, the analysis of *T*_m_ values has shown that only compound **2a** increased dsDNA stability by 7 °C in comparison to the control. For other discussed compounds (**1a**, **1b**, **2b**) the changes of melting temperatures were not statistically significant.

The proposed DNA unwinding assay has shown that only compound **2a**, at the concentration >10 µM in the presence of Topo I, converted relaxed plasmid to a negatively supercoiled molecule. Moreover, **2a** did not inhibit the overall activity of Topo I and did not stimulate DNA cleavage mediated by Topo II.

Circular dichroism spectroscopy confirmed the intercalation of compound **2a** into ct-DNA and selected ds-oligonucleotide.

In the comet assay, compound **2a** considerably reduced the effectiveness of the DNA migration, after lesion induction, which can be explained as the result of the bisintercalative binding mode of **2a** which “stapled” damaged DNA fragments (“zipper like effect”). Preliminary theoretical study has shown that interaction of the discussed molecules with dsDNA depends on molecule linker length and charge distribution over terminal aromatic chromophores.

## Figures and Tables

**Table 1:** C_50_ and *K*_app_ values for tested compounds.

Compound	* C_50_ ± SD	^†^ *K*_app_ × 10^6^
	(µM)	(M^−1^)
**1a**	52.90 ± 3.18	0.23
**1b**	104.67 ± 5.01	0.11
**2a**	2.81 ± 0.53	4.26
**2b**	11.10 ± 0.36	1.08
**9AA**	6.62 ± 0.93	1.81

* C_50_ is defined as the concentration of added compounds required to reduce the fluorescence of the DNA/EtBr complex to 50%; ^†^ *K*_app_: the apparent DNA binding constant of examined compounds. *K*_app_ was calculated as follows: *K*_app_ = 1.26/C_50_ × *K*_EtBr_, with the value of *K*_EtBr_ = 9.5 × 10^6^ M^−1^ [36].

**Table 2:** Influence of examined compounds on the thermal stability of dsDNA.

Additive	Oligonucleotide	Melting Temperature, *T*_m_ (°C)
None (negative control)	dsDNA	64.97 ± 0.20
**1a**	dsDNA	64.02 ± 0.00
**1b**	dsDNA	64.07 ± 0.14
**2a**	dsDNA	70.7 ± 0.71
**2b**	dsDNA	64.5 ± 0.71
**9AA** (positive control)	dsDNA	78.00 ± 0.00

## Supplementary Materials

Supplementary materials can be found at www.mdpi.com/1422-0067/17/9/1560/s1.

## Abbreviations

**9AA**	9-aminoacridine
B3LYP	Becke-Lee-Yang_Parr hybrid method
BSA	Bovine Serum Albumin
C_50_	the concentration of added compound required to reduce the fluorescence of the DNA/EtBr complex to 50%
DAPI	4′,6-diamidino-2-phenylindole
DFT	density functional theory
DMSO	dimethyl sulfoxide
dsDNA	double stranded Deoxyribonucleic acid
DTT	Dithiothreitol
ct-DNA	Calf thymus DNA
EDTA	Ethylenediaminetetraacetic acid (as disodium salt)
EtBr	Ethidium Bromide
GGA	generalized gradient approximation
*K*_app_	apparent binding constant of a compound
NBO	natural bond order
NPA	natural population analyses
*T*_m_	melting temperature
Topo I	topoisomerase I
Tris	Tris(hydroxymethyl)aminomethane
TBS	Tris-buffered saline
