# Supplementary material for: DNA Interaction Studies of Selected Polyamine Conjugates
Source: Int J Mol Sci. 2016 Sep 19;17(9):1560. doi: 10.3390/ijms17091560 (PMC5037830; doi:10.3390/ijms17091560)
Supplement: Supplementary file 1 [file ijms-17-01560-s001.pdf]

Supplementary Materials: DNA Interaction Studies of Selected Polyamine Conjugates

Marta Szumilak, Anna Merecz, Malgorzata Strek, Andrzej Stanczak, Tadeusz W. Inglot and Boleslaw T. Karwowski

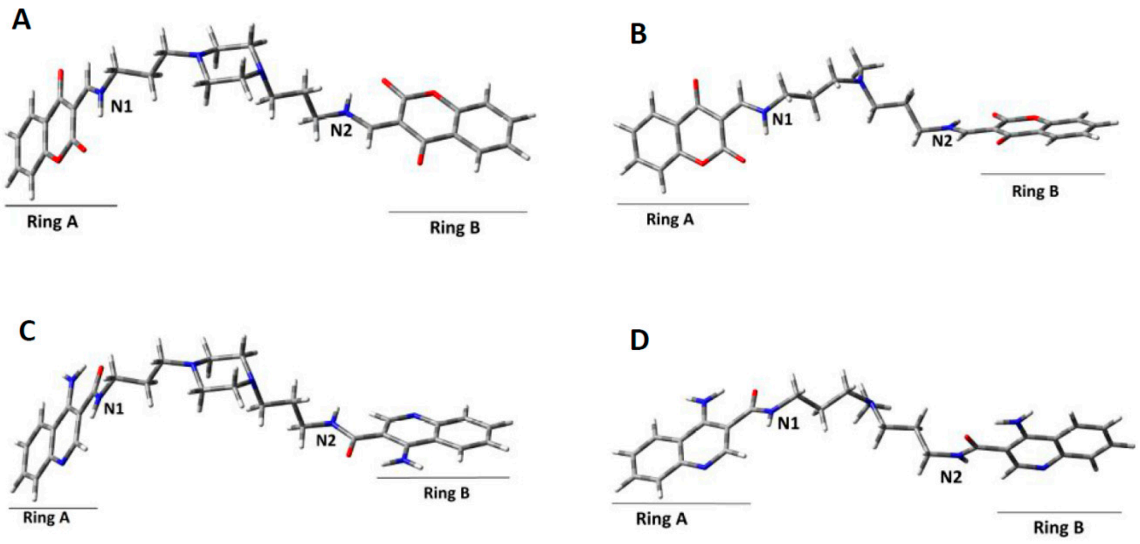

Figure S1. Spatial geometries of compounds: **1a** (**A**), **1b** (**B**), **2a** (**C**), **2b** (**D**). The structures have been optimized at the B3LYP/6-31G level of theory in the gaseous phase.

Table S1. The distances between terminal nitrogen atoms (N1 and N2) and charge distribution over aromatic rings (Ring A and B) in examined compounds obtained by calculation at the B3LYP/6-31G level of theory in the gaseous phase.

Compound	N1-N2 Distance in (Å)	Charge Distribution	
		Ring A	Ring B
**1a**	10.939	−0.6465	−0.6336
**1b**	9.271	−0.6419	−0.6366
**2a**	11.033	−0.1899	0.5998
**2b**	9.306	−0.1692	0.6179

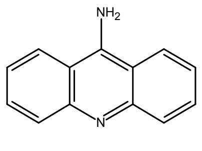

9-Aminoacridine (9-AA)

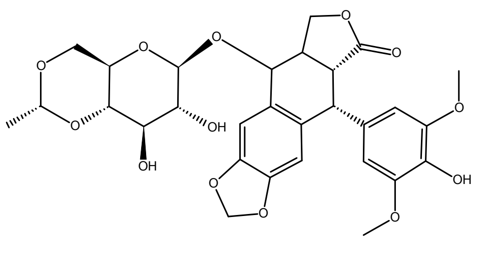

Etoposide

Figure S2. Chemical structure of reference compounds.

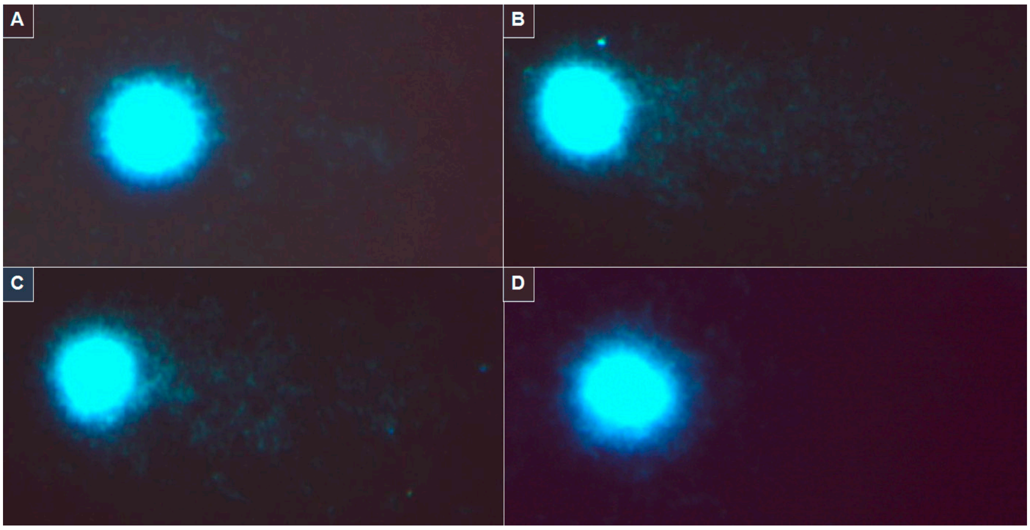

Figure S3. The representative pictures of comet assay. MOLT-4 comets from cells (control without H_2O_2 (**A**)) exposed to **2a** at the concentration of 5 μM (**B**), 10 μM (**C**) and 15 μM (**D**) after prior incubation with H_2O_2.
